# Two new fungal genera (*Diaporthales*) found on *Dipterocarpaceae* in Thailand

**DOI:** 10.3389/fmicb.2023.1169052

**Published:** 2023-06-05

**Authors:** Xia Tang, Yong-Zhong Lu, Lakmali S. Dissanayake, Ishani D. Goonasekara, Ruvishika S. Jayawardena, Yuan-Pin Xiao, Kevin D. Hyde, Xue-Mei Chen, Ji-Chuan Kang

**Affiliations:** ^1^Engineering and Research Center for Southwest Biopharmaceutical Resource of National Education Ministry of China, Guizhou University, Guiyang, Guizhou, China; ^2^Center of Excellence in Fungal Research, Mae Fah Luang University, Chiang Rai, Thailand; ^3^School of Science, Mae Fah Luang University, Chiang Rai, Thailand; ^4^School of Food and Pharmaceutical Engineering, Guizhou Institute of Technology, Guiyang, Guizhou, China; ^5^International Relations Unit, The Open University of Sri Lanka, Nawala, Nugegoda, Sri Lanka

**Keywords:** 2 new taxa, morphology, multi-gene phylogeny, saprophytic fungi, *Sordariomycetes*, taxonomy

## Abstract

*Diaporthales* is a species-rich order of fungi that includes endophytes, saprobes, and pathogens associated with forest plants and crops. They may also occur as parasites or secondary invaders of plant tissues injured or infected by other organisms or inhabit living animal and human tissues, as well as soil. Meanwhile, some severe pathogens wipe out large-scale cultivations of profitable crops, timber monocultures, and forests. Based on morphological and phylogenetic analyses of combined ITS, LSU, *tef1-α*, and *rpb2* sequence data, generated using maximum likelihood (ML), maximum parsimony (MP), and MrBayes (BI), we introduce two new genera of *Diaporthales* found in *Dipterocarpaceae* in Thailand, namely *Pulvinaticonidioma* and *Subellipsoidispora*. *Pulvinaticonidioma* is characterized by solitary, subglobose, pycnidial, unilocular conidiomata with the internal layers convex and pulvinate at the base; hyaline, unbranched, septate conidiophores; hyaline, phialidic, cylindrical to ampulliform, determinate conidiogenous cells and hyaline, cylindrical, straight, unicellular, and aseptate conidia with obtuse ends. *Subellipsoidispora* has clavate to broadly fusoid, short pedicellate asci with an indistinct J- apical ring; biturbinate to subellipsoidal, hyaline to pale brown, smooth, guttulate ascospores that are 1-septate and slightly constricted at the septa. Detailed morphological and phylogenetic comparisons of these two new genera are provided in this study.

## Introduction

*Diaporthales* is an order of ascomycetous fungi belonging to the subclass *Diaporthomycetidae* (*Sordariomycetes*) that dwell on terrestrial or aquatic taxa of plants, animals, and in soil (Senanayake et al., 2017, 2018; Wijayawardene et al., 2022). Senanayake et al. (2017, 2018) provided a recent treatment of the order and examined, described, and illustrated worldwide specimens and listed 27 families in *Diaporthales*. Many studies of this order have led to an explosion of species, including a total of 29 families (Crous et al., 2019; Guterres et al., 2019). Jiang et al. (2020) redefined the family *Cryphonectriaceae* and established two new families for the order, with a total of 31 families in *Diaporthales*. In the latest outline of the fungi and fungus-like taxa, Wijayawardene et al. (2022) accepted 32 families in the order.

*Diaporthales* contains both sexual and asexual morphs. The sexual morph is characterized by immersed stromata or substrata, brown or black perithecial ascomata with elongated beaks, sometimes with long papilla, deliquescent paraphyses at maturity, commonly unitunicate, thick-walled asci that are either evanescent with short stalks or intact, often floating free within the centrum at maturity, and have a refractive ring at the apex, containing 2–32 spores (Alexopoulus and Mims, 1978; Hawksworth et al., 1995; Castlebury et al., 2002; Rossman et al., 2007; Fan et al., 2018; Senanayake et al., 2018; Hyde et al., 2020b; Jiang et al., 2020). The asexual morph of *Diaporthales* is generally coelomycetous, rarely hyphomycetous, bearing their phialidic, rarely annellidic, conidiogenous cells, and conidia in acervuli or pycnidia with or without well-developed stromata. Since it has fewer distinguishing traits, proper identification at the genus and species levels is typically dependent on sequence data (Castlebury et al., 2002; Jiang et al., 2020).

In this study, we collected three interesting species from dead twigs and fruits of *Dipterocarpaceae* sp. from Thailand. The morphological characteristics indicated that these three taxa belong to the order *Diaporthales*. Furthermore, a phylogenetic analysis using a combination of ITS, LSU, *tef1-*α, and *rpb2* sequence data confirmed them as distinct lineages within *Diaporthales*. Therefore, two new genera named *Pulvinaticonidioma* and *Subellipsoidispora* are described herein, with detailed descriptions and illustrations.

## Materials and methods

### Sample collection, isolation, and morphological studies

Fresh samples of decaying fruits and twigs from *Dipterocarpaceae* sp. were collected at the Mushroom Research Center, Chiang Mai, Thailand, in 2019. Samples were observed using a stereomicroscope (Motic SMZ-171). The detailed method of collection, observation of specimens, and isolation were carried out as references in the study by Senanayake et al. (2020) and Tang et al. (2022). The Tarosoft (R) Image Frame Work application (IFW 0.97 version) was used to take measurements, and the photoplates were made by Adobe Photoshop CS6 (Adobe Systems, USA). The type specimens were deposited in the Mae Fah Luang University Herbarium (MFLU), Chiang Rai, Thailand, and the ex-type cultures were deposited in the Culture Collection at Mae Fah Luang University (MFLUCC). Index Fungorum (2023) and Faces of Fungi numbers were acquired as detailed by Jayasiri et al. (2015). New species are established as recommended by Chethana et al. (2021a), and the records of new taxa in the Greater Mekong Subregion were uploaded to the GMS database (Chaiwan et al., 2021).

### DNA extraction, PCR amplification, and sequencing

Fresh mycelia were prepared from the living culture that grew for 28 days and stored in the refrigerator at −20°C. DNA extraction, polymerase chain reaction (PCR) amplifications, sequencing, and phylogenetic analyses were carried out following the study by Tang et al. (2022). The manufacturer's instructions were followed while using the genomic DNA extraction kits [Sangon Biotech (Shanghai) Co., Ltd., China], in order to obtain DNA. The genes and primers used in this study were as follows: for internal transcribed spacer region (ITS), ITS5 and ITS4 (White et al., 1990); 28S large subunit rDNA region (LSU), LR0R, and LR5 (Vilgalys and Hester, 1990; Cubeta et al., 1991); translation elongation factor 1-alpha (*tef1-*α), EF1-728F, and EF2 (O'Donnell et al., 1998; Carbone and Kohn, 1999); and for RNA polymerase II second largest subunit (*rpb2*), *frpb2-5f* , and *frpb2-7cr* (Liu et al., 1999) genes. The PCR was carried out in a volume of 50 μl. The reagents that were used in the polymerase chain reaction were as follows: the DNA template (2 μl), forward primers (2 μl), reverse primers (2 μl), 2 ×Taq PCR Master Mix (25 μl), and 19 μl of ddH_2_O (double-distilled water). The annealing temperature was set to 52°C for 1 min and extension at 72°C for 90 s in LSU and ITS, followed by 35 cycles; 56°C for 1 min and extension at 72°C for 90 s in *tef1-*α, followed by 35 cycles; and 55°C for 1 min and extension at 72°C for 90 s in *rpb2* followed by 35 cycles. The products of PCR were checked on 1% agarose gels and sent to Sangon Biotech (Shanghai) Co., Ltd., China for sequencing.

### Phylogenetic analyses

The forward and reverse primers of the newly generated sequence were assembled by the Contig Express v3.0.0 application, and the most similar taxa were found by BLASTn (https://blast.ncbi.nlm.nih.gov/Blast.cgi) in NCBI. A combination of sequence data (ITS, LSU, *tef1-*α, and *rpb2*) of *Cryphonectriaceae* and *Coryneaceae* in GenBank (Tables 1, 2) was downloaded for phylogenetic analyses. Sequence data of each region were aligned by the online version of MAFFT v. 7 (https://mafft.cbrc.jp/alignment/server/index.html) (Katoh et al., 2017), through the “auto” option. Multiple genes were combined by SequenceMatrix (Vaidya et al., 2011). The aligned sequences were trimmed by manually adjusting and using trimAl v 1.2, with the “-gt 0.6” option (Capella-Gutiérrez et al., 2009). The phylogenetic analyses in this study were based on the maximum likelihood (ML), maximum parsimony (MP), and Bayesian inference (BI), by using a combined sequence dataset of ITS, LSU, *tef1-*α, and *rpb2*. The analysis of maximum likelihood (ML), maximum parsimony (MP), and Bayesian inference (BI) was processed in the CIPRES web portal (Miller et al., 2010) using the “RAxML-HPC v.8 on XSEDE” tool, “PAUP on XSEDE” tool, and “MrBayes on XSEDE” tool, respectively (Huelsenbeck and Ronquist, 2001; Swofford, 2002; Stamatakis et al., 2008; Ronquist et al., 2012).

**Table 1:** Taxa used in this study for *Cryphonectriaceae* and their GenBank accession numbers for ITS, LSU, *tef1-*α, and *rpb2* sequence data.

**Species**	**Strain number**	**GenBank accession number**			
		**ITS**	**LSU**	***tef1-**α*	***rpb2***
*Amphilogia gyrosa*	CBS 112922	AF452111	AY194107	MN271818	MN271782
*Amphilogia gyrosa*	CBS 112923	AF452112	AY194108	MN271819	MN271783
*Aurantioporthe corni*	CMW 10526	DQ120762	AF408343	NA	NA
*Aurantioporthe corni*	CBS 245.90	MN172403	MN172371	MN271822	MN271784
*Aurantiosacculus acutatus*	CBS 132181^T^	JQ685514	JQ685520	MN271823	NA
*Aurantiosacculus eucalyptorum*	CBS 130826^T^	JQ685515	JQ685521	MN271824	MN271785
*Aurantiosacculus castaneae*	CFCC 52456^T^	MH514025	MH514015	NA	MN271786
*Aurapex penicillata*	CBS 115740^T^	AY214311	AY194103	NA	NA
*Aurapex penicillata*	CBS 115742^T^	AY214313	MN172372	NA	NA
*Aurapex penicillata*	CBS 115801	MN172404	MN172373	NA	MN271787
*Aurifilum marmelostoma*	CBS 124928 ^T^	FJ890495	MH874934	MN271827	MN271788
*Aurifilum marmelostoma*	CBS 124929	FJ882855	HQ171215	MN271828	MN271789
*Celoporthe dispersa*	CBS 118782^T^	DQ267130	HQ730853	HQ730840	NA
*Celoporthe eucalypti*	CBS 127190^T^	HQ730837	HQ730863	HQ730850	MN271790
*Celoporthe guangdongensis*	CBS 128341^T^	HQ730830	HQ730856	HQ730843	NA
*Celoporthe syzygii*	CBS 127218^T^	HQ730831	HQ730857	HQ730844	NA
*Celoporthe woodiana*	CBS 118785^T^	DQ267131	MN172375	JQ824071	MN271791
*Celoporthe* sp.	CBS 534.82	MN172406	MN172376	NA	NA
*Chrysomorbus lagerstroemiae*	CBS 142594^T^	KY929338	KY929328	MN271830	NA
*Chrysomorbus lagerstroemiae*	CBS 142592	KY929330	KY929320	MN271831	NA
*Chrysoporthe austroafricana*	CBS 112916^T^	AF292041	AY194097	MN271832	NA
*Chrysoporthe austroafricana*	CBS 115843	AF273473	MN172377	MN271833	NA
*Chrysoporthe cubensis*	CBS 118654^T^	DQ368773	MN172378	MN271834	NA
*Chrysoporthe cubensis*	CBS 505.63	AY063476	MN172379	MN271835	MN271792
*Chrysoporthe hodgesiana*	CBS 115854^T^	AY692322	MN172380	MN271836	MN271793
*Chrysoporthe hodgesiana*	CBS 115744	AY956970	MN172381	MN271837	NA
*Chrysoporthe inopina*	CBS 118659^T^	DQ368777	MN172382	MN271838	NA
*Chrysoporthe syzygiicola*	CBS 124488^T^	FJ655005	MN172383	MN271839	NA
*Chrysoporthe zambiensis*	CBS 124503^T^	FJ655002	MN172384	MN271840	NA
*Corticimorbus sinomyrti*	CBS 140205^T^	KT167169	KT167179	MN271841	MN271794
*Corticimorbus sinomyrti*	CBS 140206	KT167170	KT167180	MN271842	MN271795
*Cryphonectria citrina*	CBS 109758^T^	MN172407	EU255074	MN271843	EU219342
*Cryphonectria decipens*	CBS 129351	EU442657	MN172385	MN271844	MN271796
*Cryphonectria decipens*	CBS 129353	EU442655	MN172386	MN271845	MN271797
*Cryphonectria japonica*	CFCC 52148	MH514033	MH514023	MN271846	NA
*Cryphonectria macrospora*	CBS 109764	EU199182	AF408340	NA	EU220029
*Cryphonectria neoparasitica*	CFCC 52146^T^	MH514029	MH514019	MN271847	NA
*Cryphonectria parasitica*	ATCC 38755	MH843497	MH514021	NA	DQ862017
*Cryphonectria parasitica*	CFCC 52150	AY141856	EU199123	MN271848	NA
*Cryphonectria quercus*	CFCC 52138^T^	MG866024	NA	MN271849	NA
*Cryphonectria quercicola*	CFCC 52141^T^	MG866027	NA	MN271850	NA
*Cryphonectria radicalis*	CBS 112917	AF452113	AY194101	NA	NA
*Cryptometrion aestuescens*	CBS 124007^T^	GQ369457	MN172387	MN271851	MN271798
*Cryptometrion aestuescens*	CBS 124008	GQ369458	HQ171211	MN271852	MN271799
*Diversimorbus metrosiderotis*	CBS 132866^T^	JQ862871	JQ862828	MN271857	NA
*Diversimorbus metrosiderotis*	CBS 132865	JQ862870	JQ862827	MN271858	NA
*Endothia chinensis*	CFCC 52144^T^	MH514027	MH514017	MN271860	NA
*Endothia gyrosa*	CMW 2091	AF368325	AY194114	NA	NA
*Endothia singularis*	CBS 112921	AF368323	NA	NA	NA
*Pulvinaticonidioma hyalinum*	MFLUCC 23-0002^T^	OQ747764	OQ709079	OQ750548	OQ750551
*Pulvinaticonidioma hyalinum*	MFLUCC 23-0004	OQ709075	OQ709078	OQ750547	OQ750550
*Foliocryphia eucalypti*	CBS 124779^T^	GQ303276	GQ303307	MN271861	MN271802
*Foliocryphia eucalyptorum*	CBS 142536^T^	KY979772	KY979827	MN271862	MN271803
*Holocryphia eucalypti*	CBS 115842^T^	MN172411	MN172391	MN271882	MN271804
*Holocryphia capensis*	CBS 132870^T^	JQ862854	JQ862811	MN271883	NA
*Holocryphia gleniana*	CBS 132871^T^	JQ862834	JQ862791	MN271884	NA
*Holocryphia mzansi*	CBS 132874^T^	JQ862841	JQ862798	MN271885	NA
*Immersiporthe knoxdaviesiana*	CBS 132862^T^	JQ862765	JQ862755	MN271886	MN271805
*Immersiporthe knoxdaviesiana*	CBS 132863	JQ862766	JQ862756	MN271887	MN271806
*Luteocirrhus shearii*	CBS 130776^T^	KC197021	KC197019	MN271890	MN271807
*Luteocirrhus shearii*	CBS 130775	KC197024	KC197018	MN271891	MN271808
*Microthia havanensis*	CBS 115855	DQ368735	MN172393	NA	MN271811
*Microthia havanensis*	CBS 115841	DQ368736	MN172394	NA	NA
*Microthia havanensis*	CBS 115758	DQ368737	MN172395	NA	NA
*Myrtonectria myrtacearum*	CMW 46433^T^	MG585736	MG585750	NA	NA
*Myrtonectria myrtacearum*	CMW 46435	MG585737	MG585751	NA	NA
*Rostraureum tropicale*	CBS 115725^T^	AY167435	MN172399	MN271895	MN271814
*Rostraureum tropicale*	CBS 115757	AY167438	MN172400	MN271896	MN271815
*Ursicollum fallax*	CBS 118663^T^	DQ368755	EF392860	MN271897	MN271816
*Ursicollum fallax*	CBS 118662	DQ368756	MN172401	MN271898	MN271817

Ex-type strains are indicated by “T” after the strain number, and newly generated sequences are in red.

CBS, Westerdijk Fungal Biodiversity Institute, Utrecht, the Netherlands; CFCC, China Forestry Culture Collection Center, Beijing, China; CMW, NA: not data available in Gen Bank; Forestry and Agricultural Biotechnology Institute (FABI), University of Pretoria, South Africa.

**Table 2:** Taxa used in this study for *Coryneaceae* and their GenBank accession numbers for ITS, LSU, *tef1-*α, and *rpb2* sequence data.

**Species**	**Strain number**	**GenBank accession number**			
		**ITS**	**LSU**	***tef1-**α*	***rpb2***
*Coryneum arausiaca*	MFLUCC 15-1110	MF190121	MF190067	MF377575	MF377609
*Coryneum arausiaca*	MFLUCC 13-0658	MF190120	MF190066	MF377574	MF377610
*Coryneum umbonatum*	D201	MH674329	MH674329	MH674337	MH674333
*Coryneum sinense*	CFCC 52452	MH683553	MH683561	MH685733	MH685725
*Coryneum suttonii*	CFCC 52317	MH683555	MH683563	MH685735	MH685727
*Coryneum gigasporum*	CFCC 52319	MH683557	MH683565	MH685737	MH685729
*Coryneum depressum*	D202	MH674330	MH674330	MH674338	MH674334
*Coryneum lanciforme*	D215	MH674332	MH674332	MH674340	MH674336
*Coryneum songshanense*	CFCC 52997	MK799946	MK799933	MK799822	MK799812
*Coryneum perniciosum*	CBS 130.25	MH854812	MH866313	NA	NA
*Coryneum modonium*	D203	MH674331	MH674331	MH674339	MH674335
*Coryneum castaneicola*	CFCC 52315	MH683551	MH683559	MH685731	MH685723
*Coryneum ilicis*	CFCC 52994	MK799948	MK799935	NA	NA
*Coryneum heveanum*	MFLUCC 17-0369	MH778707	MH778703	MH780881	NA
*Coryneum heveanum*	MFLUCC 17-0376	MH778708	MH778704	NA	NA
*Diaporthe eres*	MFLUCC 17-1025	KY964221	NA	KY964177	NA
*Diaporthe krabiensis*	MFLUCC 17-2481	MN047100	MN017866	MN433215	NA
*Hyaliappendispora galii*	MFLUCC 16-1208^T^	MF190150	MF190095	MF377588	NA
*Hyaloterminalis alishanensis*	NCYUCC 19-0400^T^	MT447559	MT447557	MT476042	NA
*Lamproconium desmazieri*	MFLUCC 14-1047^T^	KX430132	KX430133	NA	NA
*Lamproconium desmazieri*	MFLUCC 15-0871	KX430136	KX430137	NA	NA
*Lamproconium desmazieri*	MFLUCC 15-0872	KX430138	KX430139	NA	NA
*Neopestalotiopsis rosae*	CBS 101057	KM199359	KM116245	KM199523	MH554850
*Neopestalotiopsis protearum*	CBS 114178	LT853103	JN712564	KM199542	MH554873
*Prosopidicola albizziae*	CPC 27478	KX228274	KX228325	NA	NA
*Prosopidicola albizziae*	CBS 141298	NA	MH878213	NA	NA
*Prosopidicola mexicana*	CBS 113529	MH862932	MH874501	NA	NA
*Stegonsporium protopyriforme*	CBS 117041	EU039976	EU039992	EU040017	NA
*Stegonsporium acerophilum*	CBS 117025	EU039982	EU039993	EU040027	KF570173
*Stenocarpella macrospora*	CBS 117560	FR748048	EU754219	MG934504	NA
*Stilbospora orientali*	CBS 135075	KF570166	KF570166	KF570237	KF570197
*Subellipsoidispora guttulata*	MFLUCC 23-0003^T^	OQ709076	OQ709080	OQ750549	OQ750552
*Talekpea foeticia*	CBS 325.79^T^	MH872982	MH861215	NA	NA

Ex-type strains are indicated by “T” in superscript, and newly generated sequences are in red.

CBS, Westerdijk Fungal Biodiversity Institute, Utrecht, the Netherlands; CFCC, China Forestry Culture Collection Center, Beijing, China; MFLUCC, Mae Fah Luang University Culture Collection, Chiang Rai, Thailand; NCYUCC, NA: not data available in Gen Bank; National Chiayi University Culture Collection, Taiwan, China.

For ML analysis, the GTRGAMMA+I-Invar model of nucleotide evolution was used, and RAxML rapid bootstrapping was set to 1,000 bootstrap replicates (Stamatakis et al., 2008).

For MP analysis, 1,000 random taxa addition was used to infer trees. With branches of zero length collapsed and all multiple parsimonious trees saved, the value of Maxtrees was set to 5,000. For trees produced using various optimal criteria, parsimony score values for tree length (TL), consistency index (CI), retention index (RI), and homoplasy index (HI) were determined. To evaluate the clade stability, 1,000 iterations of the Bootstrap (BT) method were utilized, each comprising 100 trials of random stepwise addition of taxa (Hillis and Bull, 1993).

For BI, MrModeltest v2 was used for the selection of the best-fit model for each gene region. The Markov chain Monte Carlo (MCMC) algorithm was launched with four chains running concurrently from a random tree topology. When the divided frequencies' average standard deviation dropped below 0.01, the procedure was immediately terminated. The burn-in factor was set at 25%, and the sampling interval for trees was set to every 1,000th generation. The posterior probabilities (PP) for the remaining trees were computed (Dissanayake et al., 2020). Adobe Illustrator version 51.1052.0.0 and FigTree version 1.4.0 were further used to view trees (Adobe Inc., San Jose, California, United States).

## Results

### Phylogenetic analyses

**Figure 1:**
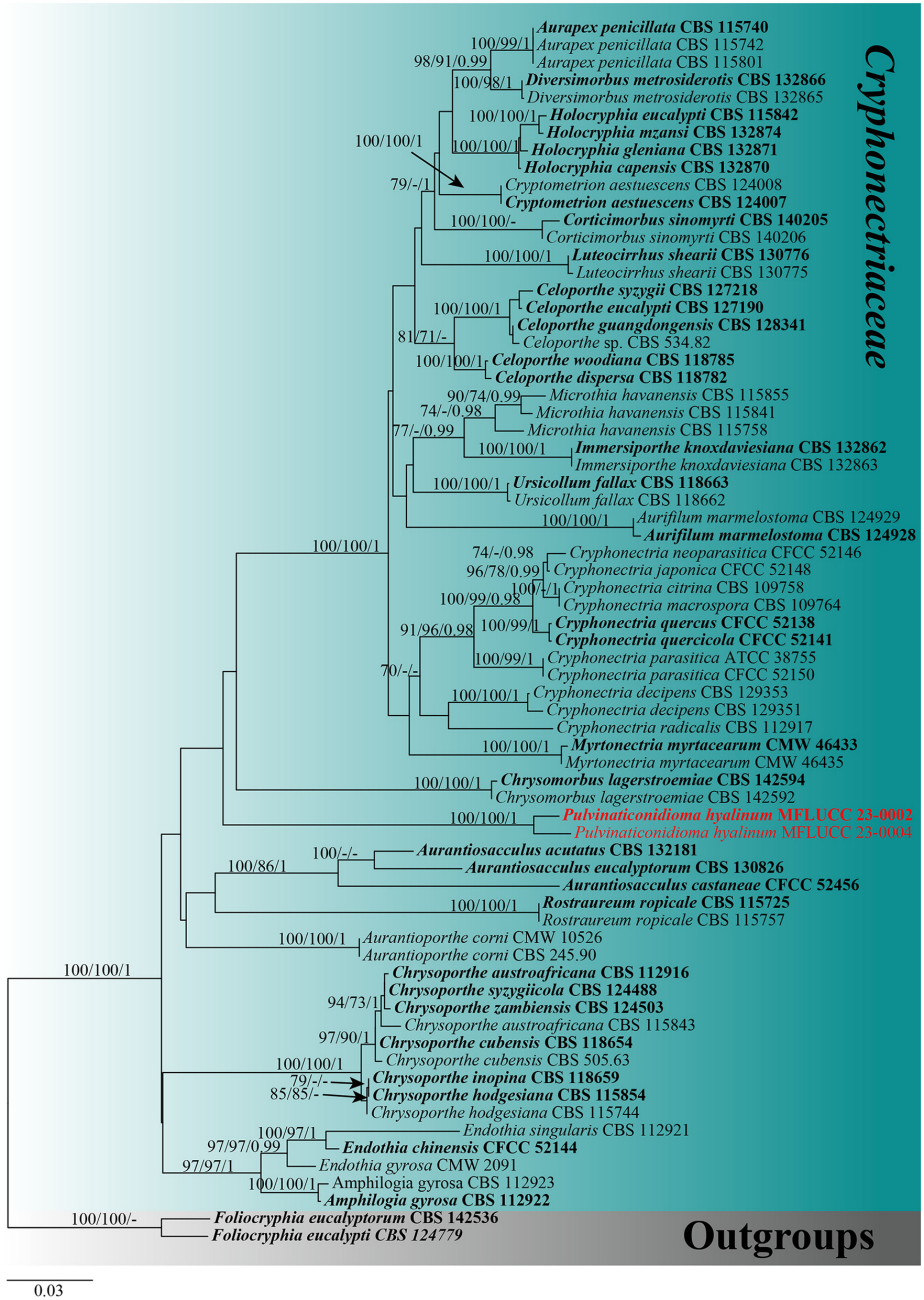
Maximum likelihood (RAxML) tree, based on the analysis of a combined dataset of ITS, LSU, *tef1-*α, and *rpb2* sequence data. The tree is rooted with *Foliocryphia eucalypti* (CBS 124779) and *Foliocryphia eucalyptorum* (CBS 142536). Bootstrap support values for ML and MP ≥70% and Bayesian posterior probabilities (BYPP) ≥0.95 are given near the nodes, respectively. Ex-type strains are in bold, and the new isolates are in red.

For the phylogenetic analyses, a combined dataset of ITS, LSU, *tef1-*α, and *rpb2* sequences was used. The dataset of *Cryphonectriaceae* included 70 taxa, with *Foliocryphia eucalypti* (CBS 124779) and *Foliocryphia eucalyptorum* (CBS 142536) as outgroups. The data matrix comprised 2,860 total characteristics, including gaps (ITS: 1–481 bp, LSU: 482–1,290 bp, *tef1-*α: 1,291–1,858 bp, and *rpb2*: 1,859–2,564 bp). Phylogenetic reconstructions with broadly comparable topologies were produced by the combined dataset of ML, MP, and BI analyses. The top-scoring ML tree with a final ML optimization likelihood value of −16,383.140512 (ln) is shown in Figure 1. In the ML analysis, the GTRGAMMA + I-Invar model was used, and the results showed 1,022 unique alignment patterns and 27.97% of indeterminate characteristics or gaps. Base frequency estimates were as follows: A = 0.229377, C = 0.266423, G = 0.271764, and T = 0.232436; substitution rates were as follows: AC = 1.760988, AG = 4.032209, AT = 1.914644, CG = 1.261342, CT = 8.527324, and GT = 1.000000; gamma distribution shape parameter alpha = 0.176927; and the tree length was 1.784127. The findings of the MP analysis showed that 2,564 characteristics remained unchanged, 103 were changeable but parsimoniously uninformative, and 733 were parsimoniously informative. The following values were displayed by the most parsimonious tree: TL = 2693, CI = 0.494, RI = 0.779, RC = 0.385, and HI = 0.506. The best-fit models for the BI analysis were GTR + I + G for ITS, LSU, *tef1-*α, and *rpb2*. With a final average standard deviation of split frequencies of 0.009895, Bayesian posterior probabilities (BYPP) from MCMC were analyzed. A new taxon correlated with the *Cryphonectriaceae* clade and is sister to *Chrysomorbus*. It is distinct from all other *Cryphonectriaceae* genera sampled herein, although with no support (Figure 1).

**Figure 2:**
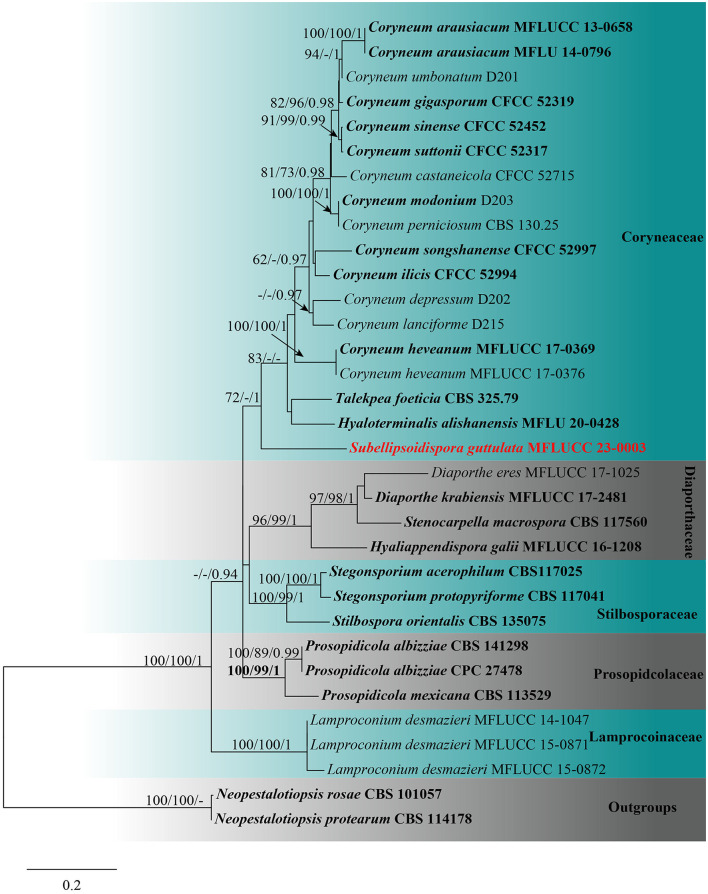
Maximum likelihood (RAxML) tree, based on the analysis of a combined dataset of ITS, LSU, *tef1-*α, and *rpb2* sequence data. The tree is rooted with *Neopestalotiopsis protearum* (CBS 114178) and *Neopestalotiopsis rosae* (CBS 101057). Bootstrap support values for ML and MP ≥70% and Bayesian posterior probabilities (BYPP) ≥0.95 are given near the nodes, respectively. Ex-type strains are in bold, and the new isolates are in red.

For the tree of *Coryneaceae*, the combined sequence dataset of 33 taxa was used with *Neopestalotiopsis protearum* (CBS 114178) and *Neopestalotiopsis rosae* (CBS 101057) as the outgroups. The data matrix comprised 2,977 total characteristics, including gaps (ITS: 1–597 bp, LSU: 598–1,426 bp, *tef1-*α: 1,427–2,123 bp, and *rpb2*: 2,124–3,151 bp). Based on the results of phylogenetic analysis, the top-scoring RAxML tree with a final ML optimization likelihood value of −19,448.697623 (ln) is shown in Figure 2. The GTRGAMMA + I-Invar model was applied to the RAxML analysis, and the findings revealed 1,332 distinct alignment patterns and 33.88% of ambiguous characteristics or gaps. The following were the base frequency estimates: A = 0.237835, C = 0.267649, G = 0.278605, and T = 0.215911; the substitution rates: AC = 1.607401, AG = 1.967526, AT = 1.403753, CG = 1.150806, CT = 5.717313, and GT = 1.000000; the gamma distribution shape parameter alpha = 0.260733; and the tree length = 3.464265. The results of the MP analysis revealed that 3,151 characteristics remained constant, 271 were variable and parsimoniously uninformative, and 1,142 were parsimoniously informative. The most frugal tree resulted in TL = 3,542, CI = 0.636, RI = 0.684, RC = 0.435, and HI = 0.364 as its values. For the BI analysis, the best-fit models were GTR+G for ITS, *tef1-*α, and *rpb2* and SYM + I + G for LSU. The BYPP from MCMC were examined with a final average standard deviation of split frequencies of 0.009847. Based on the results of phylogenetic analysis of the combined ITS, LSU, *tef1-*α, and *rpb2* sequencing data, the new taxon is related to *Coryneum, Hyaloterminalis*, and *Talekpea* within *Coryneaceae*, with statistical support of 72% ML and 1 BYPP. It differs from any other *Coryneaceae* genus sampled here (Figure 2).

### Taxonomy

***Cryphonectriaceae*** Gryzenh. & M.J. Wingf., Mycologia 98: 246. 2006.
*Index Fungorum number*: IF510585; *Facesoffungi number*: FoF03455.

**Sexual morph** see Jiang et al. (2020). **Asexual morph** *Conidiomata* semi-immersed to erumpent on the substrate, solitary, subglobose to pulvinate, pyriform, uni- to multiloculate, yellow, orange to fuscous black; necks absent or present with one to several attenuated necks. *Conidiophores* sometimes reduced to conidiogenous cells, cylindrical, hyaline, septate, or not. *Conidiogenous cells* hyaline, smooth, phialidic, ampulliform, inconspicuous, lining the inner cavity of conidiomata, with attenuate or truncate apices. *Conidia* hyaline, cylindrical, minute, seldom sigmoid, or slightly curved, aseptate (Jiang et al., 2020).

Notes: *Cryphonectriaceae* was described by Gryzenhout et al. (2006) to accommodate the *Cryphonectria*-*Endothia* complex based on LSU sequence data, and it mainly comprises plant pathogens (Vermeulen et al., 2011). Recently, Jiang et al. (2020) reevaluated this family based on morphology and combined ITS, LSU, *tef1-*α, and *rpb2* multi-gene phylogenetic analysis. It now contains 22 genera and 56 species (Jiang et al., 2020; this study).

*Type genus*: ***Cryphonectria*** (Sacc.) Sacc. & D. Sacc.

***Pulvinaticonidioma*** X. Tang, Jayaward, J.C. Kang & K.D. Hyde, gen. nov.

*Index Fungorum number*: IF900388; *Faceoffungi number*: FOF 13992
*Etymology*: The generic name refers to the pulvinate conidiomata.
*Type species*: ***Pulvinaticonidioma hyalinum*** X. Tang, Jayaward, J.C. Kang & K.D. Hyde.
*Subclass classification*: *Sordariomycetes, Diaporthales, Cryphonectriaceae*.

*Saprobic* on *Dipterocarpaceae* sp. **Sexual morph** not observed. **Asexual morph** *Coelomycetous*. *Conidiomata* immersed to semi-immersed in the substrate, solitary, glabrous or rough, pycnidial, subglobose, unilocular, thick-walled, ostiolate, brown to dark brown. *Ostiole* central, single with slightly protruding ostiolar papilla. *Conidiomata wall* composed of thick-walled, pale brown to dark brown cells of *textura angularis* at the exterior, and convex and pulvinate at the base. *Conidiophores* hyaline reduced to conidiogenous cells. *Conidiogenous cells* phialidic, cylindrical to ampulliform, determinate, smooth-walled, hyaline. *Conidia* hyaline, cylindrical, with obtuse ends, straight, unicellular, aseptate, thick- and smooth-walled.

**Figure 3:**
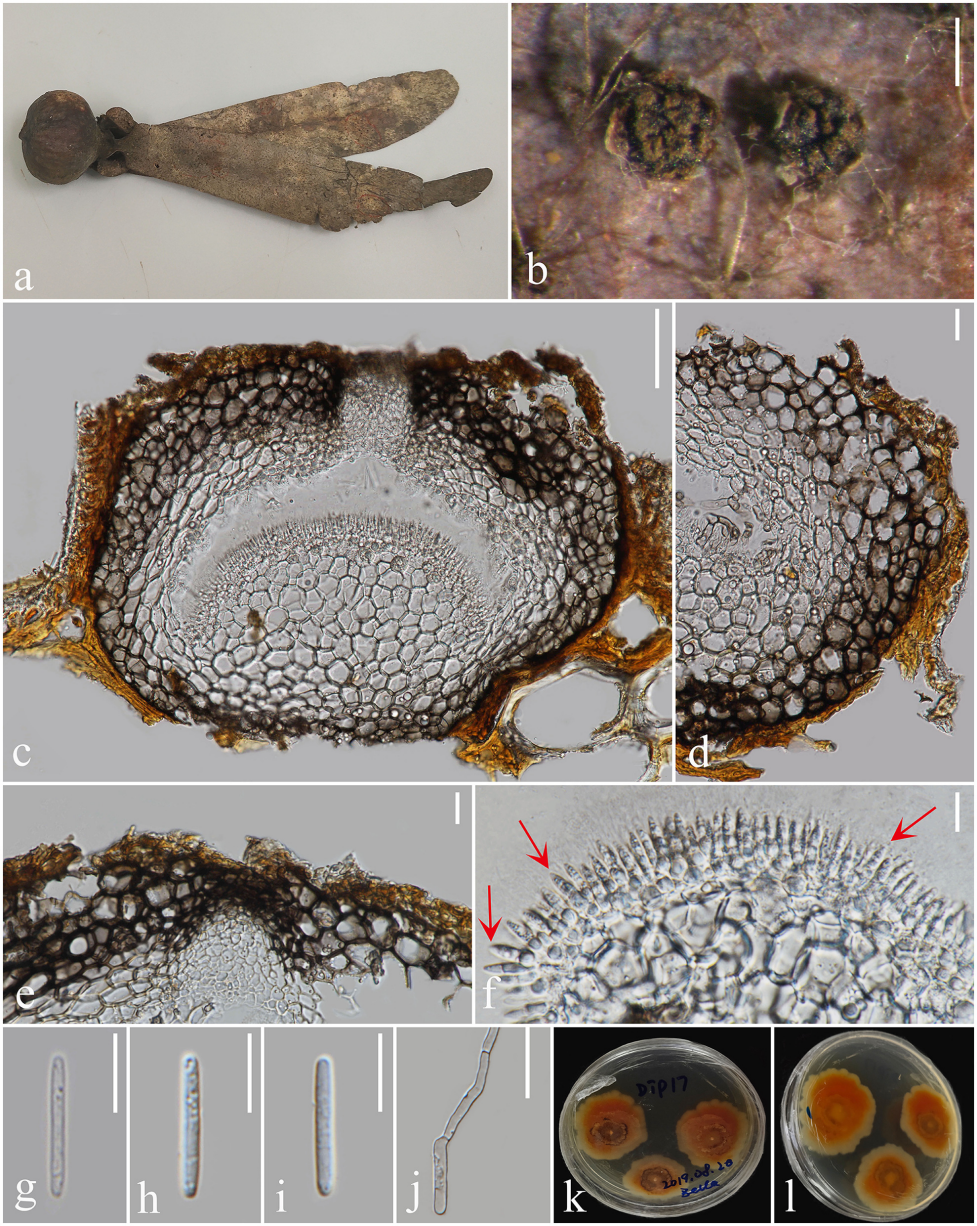
*Pulvinaticonidioma hyalinum* (MFLU 23-0052, Holotype). **(a)** Fallen pod of *Dipterocarpaceae* sp. **(b)** Conidiomata on *Dipterocarpaceae* sp. **(c)** Section of conidioma. **(d)** Conidioma wall **(e)** Ostiole. **(f)** Conidiogenous cells. **(g–i)** Conidia. **(j)** Germinated conidium. **(k)** Colonies on PDA. **(l)** Reverse of culture. Scale bars: **(b)** 500 μm, **(c, d)** 100 μm, **(e, g–j)** 20 μm, and **(f)** 10 μm.

**Figure 4:**
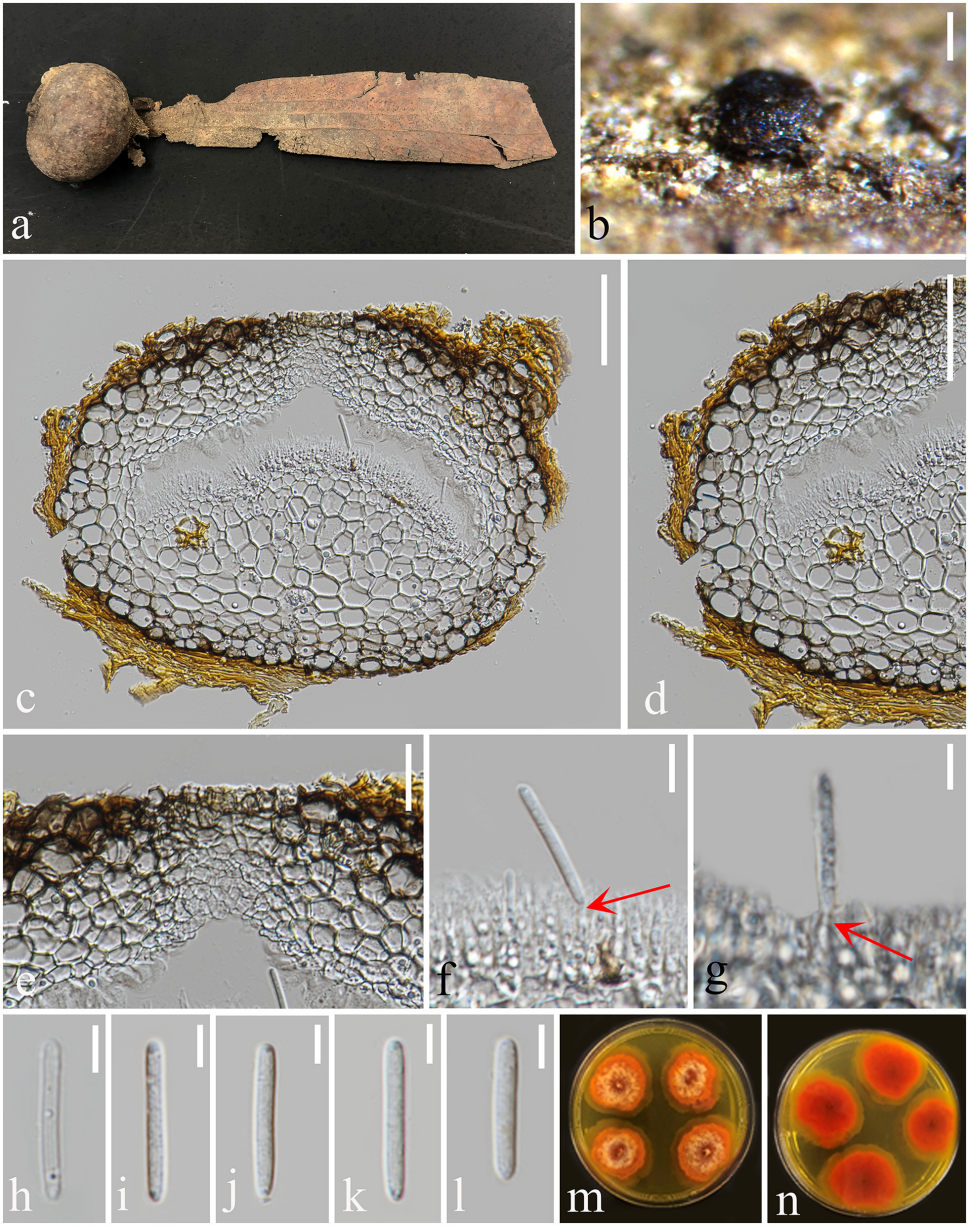
*Pulvinaticonidioma hyalinum* (MFLU 23-0053, Paratype). **(a)** Fallen pod of *Dipterocarpaceae* sp. **(b)** Conidiomata on *Dipterocarpaceae* sp. **(c)** Section of conidioma. **(d)** Conidioma wall. **(e)** Ostiole. **(f, g)** Conidiogenous cells. **(h–l)** Conidia. **(m)** Colonies from above. **(n)** The reverse of culture. Scale bars: **(b)** 200 μm, **(c, d)** 50 μm, **(e)** 20 μm, and **(f–l)** 5 μm.

*Notes: Pulvinaticonidioma* is characterized by solitary, subglobose, pycnidial conidiomata, phialidic, conidiogenous cells, and aseptate hyaline conidia. This matches with the morphological characteristics of *Cryphonectriaceae* (Jiang et al., 2020). Phylogenetically, *Pulvinaticonidioma* clusters with *Chrysomorbus* (Figures 3, 4). Both *Pulvinaticonidioma* and *Chrysomorbus* have a coelomycetous asexual morph (Chen et al., 2018). The former differs from the species in *Chrysomorbus* in having unilocular, glabrous or rough, thick-walled, ostiolate conidiomata with hyaline cells of *textura angularis* at the exterior, convex and pulvinate at the base; aseptate, straight, cylindrical, unicellular, and hyaline conidia with obtuse ends. After the comprehensive consideration based on the morphological and phylogenetic analysis, we, herein, introduce *Pulvinaticonidioma* as a new genus in *Cryphonectriaceae*, with *Pulvinaticonidioma hyalinum* as the type.

***Pulvinaticonidioma hyalinum*** X. Tang, Jayaward, J.C. Kang & K.D. Hyde, sp. nov.
*Index Fungorum number*: IF900390; *Faceoffungi number*: FOF 13993
*Etymology*: The epithet refers to the hyaline conidia.
*Holotype*: MFLU 23-0052.

*Saprobic* on *Dipterocarpaceae* sp. **Sexual morph** not observed. **Asexual morph** *Coelomycetous*. *Conidiomata* 297–473 × 211–316 μm (*x̄* = 375 × 267 μm, *n* = 20), immersed to semi-immersed in substrate, solitary, glabrous or rough, pycnidial, subglobose, unilocular, thick-walled, ostiolate, brown to dark brown. *Ostiole* 51–65 × 34–48 μm (*x̄* = 58 × 42 μm, *n* = 10), central, single with slightly protruding ostiolar papilla. *Conidiomata wall* 50–88 μm (*x̄* = 70 μm, *n* = 20) wide, composed of thick-walled, pale brown to dark brown cells of *textura angularis* at the exterior, convex and pulvinate at the base 103–202 μm high (*x̄* = 144 μm, *n* = 20). *Conidiophores* hyaline, reduced to conidiogenous cells. *Conidiogenous cells* 6–11.5 × 1.8–3.4 μm (*x̄* = 7 × 2.5 μm, *n* = 20), phialidic, cylindrical to ampulliform, determinate, smooth-walled, hyaline. *Conidia* 15–20 × 2–3 μm (*x̄* = 17 × 2.5 μm, *n* = 20) hyaline, cylindrical, with obtuse ends, straight, unicellular, aseptate, thick- and smooth-walled.

*Culture characters*: *Conidia* germinated on PDA within 24 h, and germ tubes produced from one end. The culture was incubated at room temperature. Colonies reached 45 mm diameter after 15 days, flat, spreading, fluffy colonies, circular with irregular lightly orange outer ring, cottony. The surface is lightly rough, with orange-red colonies, cream-colored hyphae attached to the center of the colony, with an irregular orange-yellow edge. The reverse orange-red, more orange-yellow at the margins, not pigmented.

*Material examined*: Thailand, Chiang Mai province, Mae Taeng District, on the fruits (pericarp and wings of the pod) of *Dipterocarpaceae*, 8 August 2019, Xia Tang, Dip17 (MFLU 23-0052, holotype; ex-type living culture, MFLUCC 23-0002), on the fruits of *Dipterocarpaceae*, 23 October 2020, Xia Tang, Dip41 (MFLU 23-0053, paratype; ex-paratype living culture, MFLUCC 23-0004).

*Notes*: The two *Pulvinaticonidioma hyalinum* collections, showing similar morphology clustered together with ML = 100, MP = 100, and BYPP = 1 support (Figure 1). The base pair differences between the two strains were as follows: ITS = 0.7% (4/557), LSU = 0% (0/811), *tef1-*α = 6.2% (38/613), and *rpb2* = 1% (11/983), respectively, and we identified them as the same species following the guidelines for species delineation proposed by Chethana et al. (2021a). *Pulvinaticonidioma hyalinum* matches the characteristics of *Cryphonectriaceae* and is similar in having unilocular conidiomata without necks and conidiomata walls made of cells of *textura globulosa* (Jiang et al., 2020). However, *P. hyalinum* differs from the type species of *Cryphonectriaceae, Chrysomorbus lagerstroemia*e in their fruiting body, conidiomata wall, conidiophores, and conidia. *Pulvinaticonidioma hyalinum* has brown to dark brown conidiomata with slightly protruding ostiolar papilla, hyaline cells of *textura angularis* at the exterior, interior layers that are convex and pulvinate at the base, and unbranched conidiophores, while *Ch. lagerstroemiae* has uni- to multilocular, conidiomata lacking ostioles, with convoluted locules, and occasionally aseptate conidia with separating septa and branching conidiophores. The conidiogenous cells in *P. hyalinum* are phialidic, cylindrical to ampulliform with hyaline, straight, aseptate, unicellular, conidia with obtuse ends, while *Ch. lagerstroemia*e has flask-shaped conidiogenous cells with attenuated apices and minute, cylindrical conidia with obtuse ends, that are hyaline, fusoid to oval, aseptate, and exuded as orange droplets (Chen et al., 2018). The phylogenetic analysis of the combined ITS, LSU, *tef1-*α, and *rpb2* sequence data showed that *P. hyalinum* belongs to *Cryphonectriaceae* and forms a separate lineage sister to *Chrysomorbus*. Although the bootstrap values are low, the phylogenetic analysis supports the placement of our new taxa in *Cryphonectriaceae*, as well as the possibility of other close relatives that have not yet been discovered; hence, their placement within the family is subjected to change. The base pair differences between *P. hyalinum* and the type species of *Chrysomorbus, viz. Ch. lagerstroemiae* were as follows: ITS = 5% (27/539), LSU = 1.4% (11/811), and *tef1-*α = 26.5% (151/569), respectively. Based on the phylogenetic analysis and morphological comparison of the nearest genus, we, herein, introduce *Pulvinaticonidioma* as a new genus to accommodate the new collection, *P. hyalinum*.

***Coryneaceae*** Corda, Icon. fung. (Prague) 3: 36 (1839) amend.
*Index Fungorum number*: IF80650; *Facesoffungi number*: FoF06868;

*Saprobes* and *pathogens* exist on dead wood and living plants, respectively. **Sexual morph:** *Stromata* erumpent, solitary, comprising pseudoparenchymatous cells. *Ectostromatic* comprising small cells of *textura prismatica*, brown to black, disk well or poorly developed. *Ascomata* brown to black, ostiolate, aggregated, immersed, arranged in valsoid configuration, perithecial, coriaceous, globose to subglobose, papillate. *Papilla* central, upright, sometimes converging, broad, comprising brown cells of *textura porrecta*. *Peridium* thick-walled, comprising outer, brown cells of *textura angularis* and inner, thick-walled, hyaline, compressed cells of *textura angularis*. *Paraphyses* attached to the base, cellular, broad, septate, longer than asci. *Asci* ellipsoid to cylindrical, unitunicate, 8-spored, pedicellate, rounded at the apex with a J-, apical ring. *Ascospores* hyaline or initially hyaline, brown at maturity, overlapping uni- to biseriate, irregularly fasciculate, ellipsoid, 1–3-septate, fusoid or elongate, sometimes end-cells pointed, often distoseptate, pale brown or hyaline end-cells, straight or curved not constricted at the septa, guttulate, smooth-walled (added from Hyde et al., 2020b). **Asexual morph:** see Hyde et al. (2020b) and Rathnayaka et al. (2020).

*Type genus*: ***Coryneum*** Nees

*Notes*: *Coryneaceae* was described by Corda (1839) to accommodate *Coryneum* as the type genus. Rathnayaka et al. (2020) amended the description of this family to accommodate these genera based on their morphological characteristics and treated *Talekpea* and *Hyaloterminalis* in *Coryneaceae*. Until now, there are three genera included in *Coryneaceae, viz. Coryneum* (Nees von Esenbeck, 1816), *Hyaloterminalis*, and *Talekpea* (Rathnayaka et al., 2020; Wijayawardene et al., 2022).

***Subellipsoidispora*** X. Tang, Jayaward, J.C. Kang & K.D. Hyde, gen. nov.
*Index Fungorum number*: IF900389; *Faceoffungi number*: FOF 13994
*Etymology*: The epithet refers to the subellipsoidal ascospores.
*Type species:* ***Subellipsoidispora guttulata*** X. Tang, Jayaward, J.C. Kang and K.D. Hyde
*Subclass classification: Sordariomycetes, Diaporthales, and Coryneaceae*.

*Saprobic* on *Dipterocarpaceae* sp. **Asexual morph** Not observed. **Sexual morph** *Ascomata* perithecial, erumpent, scattered, solitary, coriaceous, immersed, globose to subglobose, papillate, ostiolate, dark brown to black. The *Ostiole* canal narrowing toward the base, internally covered by hyaline periphyses, cells around the base small, thick-walled, and brown. *Peridium* comprising brown, compressed, cells of *textura angularis*. *Hamathecium* composed of cylindrical, unbranched, straight to flexible, smooth, hyaline, septate paraphyses slightly constricted at the septa, tapering toward to end, longer than asci. *Asci* 8-spored, unitunicate, clavate to broadly fusoid, short pedicellate, apex blunt, with an indistinct, J- apical ring, evanescent. *Ascospores* overlapping uniseriate to biseriate, biturbinate to subellipsoidal, 1-septate, slightly constricted at the septa, guttulate, smooth, hyaline to pale brown.

**Figure 5:**
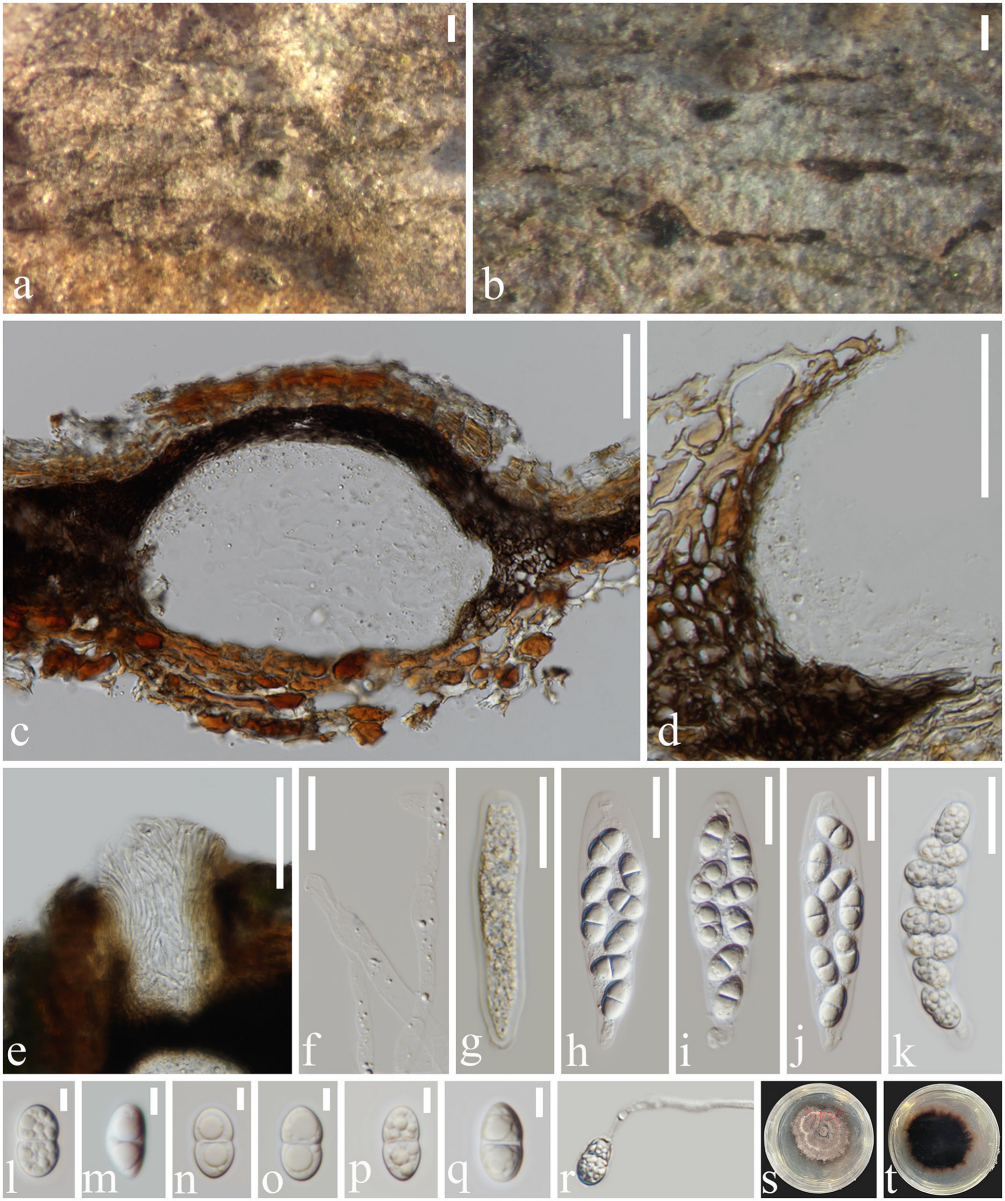
*Subellipsoidispora guttulata* (MFLU 23-0054, holotype) **(a, b)** Appearance of ascomata on host substrate. **(c)** Section of an ascoma. **(d)** Peridium. **(e)** Ostiole. **(f)** Paraphyses. **(g–k)** Asci from immature to mature. **(l–q)** Ascospores. **(r)** Germinated ascospore. **(s)** Colony on PDA. **(t)** The reverse of culture. Scale bars: **(a, b)** 200 μm, **(c–e)** 50 μm, **(f–k)** 20 μm, and **(l–q)** 5 μm.

*Notes*: *Subellipsoidispora* share characteristics with *Coryneaceae*, such as perithecial, coriaceous, ostiolate, brown to black ascomata; with thick-walled peridium having outer and inner brown cells of *textura angularis* and hyaline, compressed cells of *textura angularis*, respectively; paraphyses are longer than asci; clavate to broadly fusoid, 8-spored asci with J- apical ring; guttulate and smooth, hyaline to pale brown and straight ascospores (Hyde et al., 2020b). *Coryneaceae* contains three genera, *viz*. *Coryneum, Hyaloterminalis*, and *Talekpea* (Rathnayaka et al., 2020). Both *Subellipsoidispora* and *Coryneum* have the ascomycetous sexual morph, while *Talekpea* and *Hyaloterminalis* have a hyphomycetous asexual morph (Senanayake et al., 2017, 2018). *Subellipsoidispora* differs from the species in *Coryneum* in having scattered, solitary ascomata; a thick-walled ostiolar canal narrowing toward the base, internally covered by hyaline periphyses, a peridium of brown-walled, compressed, cells of *textura angularis*, clavate to broadly fusoid, short pedicellate asci and biturbinate to subellipsoidal, 1-septate, guttulate ascospores, slightly constricted at the septa. In the phylogenetic analysis, *Subellipsoidispora* clusters in *Coryneaceae* and forms a separate lineage sister to *Hyaloterminalis* and *Talekpea* (Figure 2). Based on its unique morphology (Figure 5) and phylogenetic evidence (Figure 1), *Subellipsoidispora* is introduced as a new genus of *Coryneaceae*, and the sexual morph is described in this study, awaiting the discovery of its asexual morph.

***Subellipsoidispora guttulata*** X. Tang, Jayaward, J.C. Kang & K.D. Hyde, sp. nov.
*Index Fungorum number*: IF900391; *Faceoffungi number*: FOF 13995
*Etmology*: Name referring to the hyaline ascospores.
*Holotype:* MFLU 23-0054.

*Saprobic* on dead barks of *Dipterocarpaceae* sp. **Sexual morph** *Ascomata* 117–270 × 71–155 μm (*x̄* = 199 × 105 μm, *n* = 20), immersed, scattered, solitary, globose to subglobose, dark brown to black, coriaceous, ostiolate, papillate. The *Ostiole* canal narrowing toward the base, internally covered by hyaline periphyses, cells around the base small, thick-walled, and brown. *Peridium* 8–28 μm wide (*x̄* = 18 μm, *n* = 20), comprising brown, compressed, cells of *textura angularis*. *Paraphyses* 3–6 μm wide (*x̄* = 5.5 μm, *n* = 30), cylindrical, unbranched, straight to flexible, smooth, hyaline, septate, slightly constricted at the septa, tapering toward to end, longer than asci. *Asci* 67–90 × 13–24 μm (*x̄* = 79 × 19 μm, *n* = 20), 8-spored, unitunicate, clavate to broadly fusoid, short pedicellate, apex blunt, with an indistinct, J- apical ring, evanescent. *Ascospores* 13–16 × 5–9 μm (*x̄* = 14 × 7 μm, *n* = 20), overlapping uniseriate to biseriate, biturbinate to subellipsoidal, 1-septate, slightly constricted at the septa rounded at both ends, guttulate, smooth-walled, hyaline to pale brown. **Asexual morph** not observed.

*Culture characters*: Colonies grown on PDA and incubated at 25°C reached a diameter of 40 mm after 2 weeks, flat, spreading, fluffy, with a pale brown ring interlaced in the colonies. Surface lightly rough with brown mycelium, colonies somewhat raised in the middle, and with an irregular edge. The reverse side dark brown with an irregular, penniform, brown edge, and not pigmented.

*Material examined*: Thailand, Chiang Mai Province, Mae Taeng district, on dead bark of *Dipterocarpaceae*, 15 July 2020, Xia Tang, Dip25 (MFLU 23-0054, holotype; ex-type living culture, MFLUCC 23-0003).

*Notes*: *Subellipsoidispora guttulata* is similar to *Coryneum umbonatum* in having immersed, coriaceous, brown to black ascomata, and unitunicate asci with an indistinct J- apical ring. However, *S. guttulata* differs from *C. umbonatum* in having clavate to broadly fusoid, short pedicellate asci and subellipsoidal, 1-septate, guttulate, hyaline to pale brown ascospores, while *C. umbonatum* has ellipsoid to cylindrical, stalk pedicellate asci, and ellipsoid, fusoid or elongate, distoseptate, straight or curved spores that are brown at maturity (Senanayake et al., 2018). Phylogenetic analysis showed that *S. guttulata* belongs to *Coryneaceae* and forms a basal lineage sister to *Coryneum*, an ascomycetous genus, *Hyaloterminalis* and *Talekpea*, a hyphomycetous and monotypic genus. The base pair differences between *S. guttulata* and *C. umbonatum* were as follows: ITS = 7.7% (45/581), LSU = 3.2% (26/842), and *rpb2* = 21.7% (223/1029), and the differences between *S. guttulata* and *Talekpea foeticia* were as follows: ITS = 12.5% (65/520) and LSU = 2% (17/843). Based on its phylogenetic and morphological analyses, we place *S. guttulata* as the type species of *Subellipsoidispora* in *Coryneaceae*.

## Discussion

*Diaporthales* (*Sordariomycetes*) is an order that contains saprobic, endophytic, and pathogenic taxa with a wide distribution on a variety of hosts (Barr, 1978; Castlebury et al., 2002; Rossman et al., 2007; Senanayake et al., 2017, 2018; Fan et al., 2018; Jiang et al., 2020). The pathogenic members cause great economic losses, such as chestnut blight, caused by *Cryphonectria parasitica* (*Cryphonectriaceae*) (Gryzenhout et al., 2006; Rigling and Prospero, 2018; Gomdola et al., 2022), polar and willow canker on *Populus* and *Salix*, caused by *Cytospora chrysosperma* (*Cytosporaceae*) (Fan et al., 2014, 2020; Wang et al., 2015), and stem-end rot of citrus fruits infected by *Diaporthe citri* (Huang et al., 2013). Researchers have carried out their research on secondary metabolites in *Diaporthaceae* and *Gnomoniaceae* (Chepkirui and Stadler, 2017; Wu et al., 2019). As saprobes, they cause the degradation of wood, such as *Apiosporopsis carpinea* (*Apiosporopsidaceae*) on the overwintered leaves of *Carpinus betulus* (Senanayake et al., 2017) and *Pseudoplagiostoma dipterocarpicola* on the decaying wood of *Dipterocarpaceae* (Tang et al., 2022). As endophytes, they live in medicinal plants and are used for studies that investigate antimicrobial activities, e.g., *Diaporthe* spp., which were isolated from the hosts *Copaifera langsdorffii* and *C. pubiflora* (de Carvalho et al., 2021). Antibacterial activity has been demonstrated using extracts of two unidentified *Diaporthe* spp. and *D. miriciae* (Carvalho et al., 2018).

As more taxonomic studies of fungi are being conducted, the focus has steadily shifted from morphology to a combination of molecular phylogeny and morphology, serving as the foundation for the mainstream approach (Senanayake et al., 2017, 2018; Jiang et al., 2020; Chethana et al., 2021a; Maharachchikumbura et al., 2021). Initially, Castlebury et al. (2002) accepted *Cytosporaceae, Diaporthaceae, Gnomoniaceae*, and *Melanconidaceae* in *Diaporthales* by using LSU sequence data. Réblová et al. (2004) established a new family *Togniniaceae* to accommodate *Togninia* and its *Phaeoacremonium* anamorphs using LSU and SSU sequence data. Later, the family *Togniniaceae* was transferred into *Togniniales* from *Diaporthales* using LSU, SSU, *tef1-*α, and *rpb2* sequence data (Gramaje et al., 2015; Maharachchikumbura et al., 2015, 2016). The use of multi-gene analysis for the identification of *Diaporthales* species was seen in subsequent studies, such as the combination of ITS-beta-tubulin (*tub2*) and ITS-LSU (Gryzenhout et al., 2006; Mostert et al., 2006; Cheewangkoon et al., 2010; Crous et al., 2012; Voglmayr et al., 2012, 2017; Suetrong et al., 2015; Réblová et al., 2016; Du et al., 2017; Yang et al., 2018; Maharachchikumbura et al., 2021). Voglmayr and Jaklitsch (2014) demonstrated through the evaluation of *Stegonsporium* and *Stilbospora* that LSU alone did not always contain sufficient phylogenetic resolution to identify consistently well-supported phylogenetic relationships at the generic level, and our research results matched this as well. Subsequently, *Schizoparmaceae* was revised using a combination of LSU, *rpb2*, ITS, and *tef1-*α (Alvarez et al., 2016). Combining DNA sequence data of ITS, LSU, *tef1-*α, and *rpb2* is advised by Senanayake et al. (2017, 2018) and Fan et al. (2018) to evaluate the phylogenetic relationships of diaporthalean families. Jiang et al. (2020) used the combination of ITS, LSU, *tef1-*α, and *rpb2* to redefine the family *Cryphonectriaceae* and to describe two new families, *viz*. *Foliocryphiaceae* and *Mastigosporellaceae*. With the increasing number of studies and knowledge on the diversity of lifestyles in *Diaporthales*, identifying its species has become difficult. The utilization of protein genes makes it possible to have a precise placement in *Diaporthales*, as proven in recent studies (Senanayake et al., 2017, 2018; Jiang et al., 2020). Thus, we suggest analyzing the families in *Diaporthales via* both morphological and molecular traits and the specific genes of each family for multigene phylogenetic analysis.

Members of the *Dipterocarpaceae* are economically significant trees generating lumber, camphor, and resin and are common in Southeast Asia (Maury-Lechon and Curtet, 1998). In this study, two new genera, namely *Pulvinaticonidioma* and *Subellipsoidispora*, were found on *Dipterocarpaceae* species in Thailand and were introduced. We introduce our collections as new genera based on unique features, such as the characteristics of the conidiomata, conidiogenous cells, and conidial appearance, as observed in the new taxon, *Pulvinaticonidioma hyalinum* when compared with other known genera in *Cryphonectriacea*. The results of the ML, MP, and MrBayes analyses also support that this is a new genus in *Cryphonectriaceae* (Figure 1). Similarly, the second collection *Subellipsoidispora guttulata* is morphologically distinct from other known genera in *Coryneaceae* in having unique characteristics in their asci and the shape of ascospores, and the phylogeny supports it as a new genus in *Coryneaceae* (Figure 2). To date, eight species of microfungi on *Dipterocarpaceae* have been described from Thailand, *viz*. *Hermatomyces thailandica, Lauriomyces sakaeratensis, Lembosia xyliae, Pseudoplagiostoma dipterocarpi, P. dipterocarpicola, Pestalotiopsis shoreae, Pulvinaticonidioma hyalinum*, and *Subellipsoidispora guttulata* (Suwannarach et al., 2016; Chethana et al., 2021b; Farr and Rossman, 2022; Tang et al., 2022; This study). Among these species, *Pseudoplagiostoma dipterocarpi* is an endophyte, while the rest are saprobes. It is remarkable that in this study, we found two new genera in a family that has been relatively well studied but on lesser studied hosts. This indicates that many more taxa will be discovered with further surveys on *Dipterocarpaceae* and other poorly studied hosts (Hyde et al., 2020a; Bhunjun et al., 2022).

## Data availability statement

The datasets presented in this study can be found in online repositories. The names of the repository/repositories and accession number(s) can be found in the article/supplementary material.

## Author contributions

XT conducted the experiments, analyzed the data, and wrote the manuscript. Y-ZL, RJ, KH, and J-CK planned the experiments. XT and LD analyzed the data. XT and X-MC conducted the experiments. Y-ZL, RJ, KH, LD, IG, Y-PX, and J-CK revised the manuscript. Y-ZL, KH, and J-CK funded the experiments. All authors revised and agreed to the published version of the manuscript.
